# Accuracy of cardiovascular magnetic resonance in myocarditis: comparison of MR and histological findings in an animal model

**DOI:** 10.1186/1532-429X-12-49

**Published:** 2010-08-26

**Authors:** Huedayi Korkusuz, Philip Esters, Frank Huebner, Reinhold Bug, Hanns Ackermann, Thomas J Vogl

**Affiliations:** 1 Department of Diagnostic and Interventional Radiology, Johann Wolfgang Goethe University, Theodor-Stern-Kai 7, 60590 Frankfurt, Germany; 2 Senckenberg Institute of Pathology, Johann Wolfgang Goethe University, Theodor-Stern-Kai 7, 60590 Frankfurt, Germany; 3 Department of Biomathematics, Johann Wolfgang Goethe University, Theodor-Stern-Kai 7, 60590 Frankfurt, Germany

## Abstract

**Background:**

Because Endomyocardial Biopsy has low sensitivity of about 20%, it can be performed near to myocardium that presented as Late Gadolinium Enhancement (LGE) in cardiovascular magnetic resonance (CMR). However the important issue of comparing topography of CMR and histological findings has not yet been investigated. Thus the current study was performed using an animal model of myocarditis.

**Results:**

In 10 male Lewis rats Experimental Autoimmune myocarditis was induced, 10 rats served as control. On day 21 animals were examined by CMR to compare topographic distribution of LGE to histological inflammation. Sensitivity, specificity, positive and negative predictive values for LGE in diagnosing myocarditis were determined for each segment of myocardium. Latter diagnostic values varied widely depending on topographic distribution of LGE and inflammation as well as on the used CMR sequence. Sensitivity of LGE was up to 76% (left lateral myocardium) and positive predictive values were up to 85% (left lateral myocardium), whereas sensitivity and positive predictive value dropped to 0-33% (left inferior myocardium).

**Conclusions:**

Topographic distribution of LGE and histological inflammation seem to influence sensitivity, specifity, positive and negative predictive values. Nevertheless, positive predictive value for LGE of up to 85% indicates that Endomyocardial Biopsy should be performed "MR-guided". LGE seems to have greater sensitivity than Endomyocardial Biopsy for the diagnosis of myocarditis.

## Background

The diagnosis of acute myocarditis is still challenging as its clinical presentation is variable. It can mimic acute myocardial infarction with symptoms of acute heart failure as well as present as silent course [1]. Elevated biochemical markers (Troponin, Creatin-Kinase, etc.), ischemia-like ECG-changes and segmental wall motion abnormalities may not be specific for acute myocarditis in every case[1]. Thus, safely diagnosing myocarditis is bound to Endomyocardial Biospy (EMB, gold standard). As sensitivity of EMB alone is low (about 20%[2]), it can combined with cardiovascular magnetic resonance (CMR) to reveal areas of myocardial inflammation. Afterwards biopsies are taken from myocardium presenting as Late Gadolinium Enhancement (LGE) to increase sensitivity of EMB[3,4].

To date the important issue of comparing the topographic distribution of LGE to the histologically proven topographic distribution of myocardial inflammation in the whole myocardium has not been investigated. Most clinical studies compared CMR-findings to Endomyocardial Biopsy, thus data concerning sensitivity and positive predictive value for LGE in CMR, validated in animal model of myocarditis with the possibility to examine the whole myocardium is missing. Experimental Autoimmune Myocarditis (EAM) in a rat model is an established animal model to display acute human myocarditis[5-8] as its histomorphology is similar to human myocarditis[5,9,10].

The main aim of the current study was to evaluate, if areas of LGE have identical topographic distribution as compared to histologically proven areas of inflammation; further to explore possible correlations between LGE and serology (troponinT, haptoglobin and proBNP) and between LGE and histological severity of myocarditis.

## Methods

### Animals

Twenty male Lewis rats (Charles River, Sulzfeld, Germany), aged 6-8 weeks, weighing 250-300 g were used in this study. Animals were randomized to control (n = 10) and to experimental group (n = 10). Animals were kept under standard conditions with a mean temperature of 22°C ± 2°C, a mean relative humidity of 55% ± 10 and a defined day-and-night-rhythm of 12 h light and 12 h dark. The study was approved by the governmental committee and our institutional animal research review board.

### Induction of EAM

Induction of EAM was performed as described before [11]. Briefly, Porcine Cardiac Myosin (Antigen; PCM M0531, Sigma Aldrich, Schnelldorf, Germany) was diluted with phosphate-buffered saline and finally emulsified with Complete Freud's Adjuvance (CFA, BD, Heidelberg, Germany). Animals of the experimental group were subcutaneously injected with 0.5 ml of the PCM-CFA-emulsion in the footpad on days 1 and 7. Animals of the control group were subcutaneously injected on days 1 and 7 with 0.25 ml CFA alone.

### Serological parameters

On days 1, 7 and 21 the retrobulbar venous plexus of all animals was punctured, 0.5 ml blood was extracted and serological levels of troponinT, NT-proBNP and haptoglobin were determined. In humans as well as in rodents troponinT is a sensitive marker for cardiac damage[12] whereas haptoglobin seems to be a more sensitive marker for unspecific inflammation comparable to C-reactive Protein (CRP) used in humans[13].

### CMR protocol

All animals were examined by clinical CMR (1.5T Magnetom Sonata, Siemens, Erlangen, Germany) using a human finger-surface-coil as receiver fixed on the animals chest on day 21. The value of such coil is to maximise the signal to noise ratio (SNR), as the SNR for a coil drops as a function of the square of the distance between the coil and tissue. Consequently, this increases the sensitivity of image analysis [14]. All CMR examinations were performed under isoflurane anesthesia (2.2% isoflurane in air) due to its negative inotropic effect[15].

As recommended in literature[14] inversion time for ECG-triggered Inversion-Recovery Turbo-FLASH-sequences (IR-Turbo-FLASH) was determined 10 minutes after intravenous injection of Gadolinium-DTPA (0.1 mmol/kg) for each examined animal, resulting in individual inversion times (range 150-280 ms). Fifteen minutes after Gadolinium injection propofol and metoprolol were injected to reduce heart rate to 90-120 bpm and CMR-examination started with ECG-triggered IR-Turbo-FLASH-sequences (FoV read 120 mm/FoV phase 46.0%/Slice thickness 3 mm/TR 750 ms/TE 4.38 ms/Averages 2/Flip angle 25°/Base resolution 256/Phase resolution 100%) first without and directly afterwards with fat saturation.

Following IR-Turbo-FLASH-examination, cardiac arrest was achieved by overdosing propofol and Turbo-Spin-Echo-Sequences (TSE) (FoV read 100 mm/FoV phase 57.8%/Slice thickness 2 mm/TR 742 ms/TE 17 ms/Averages 3/Flip angle 180°/Fat saturated/base resolution 448/Phase resolution 100%) with a higher signal-to-noise-ratio were performed, first without and directly afterwards with fat saturation.

### CMR-Analysis

Two radiologists, who were blinded to the results of histopathological examination, with 5 and 8 years experience in CMR analysed MR images in consensus. Areas of LGE were traced by manually drawing regions of interest (ROI) using a commercially available PACS-System (Centricity, GE Health Care Products and Solutions, Chicago, USA). For each CMR-sequence (Turbo-FLASH, TSE, with/without fat saturation) in three representative slices from heart base to apex the area of myocardium that showed LGE was calculated as a percentage of the whole surface area of the myocardium in the respective slice; percentages of LGE-areas were summed up for the respective CMR-sequence and for each animal separately.

**Figure 1:**
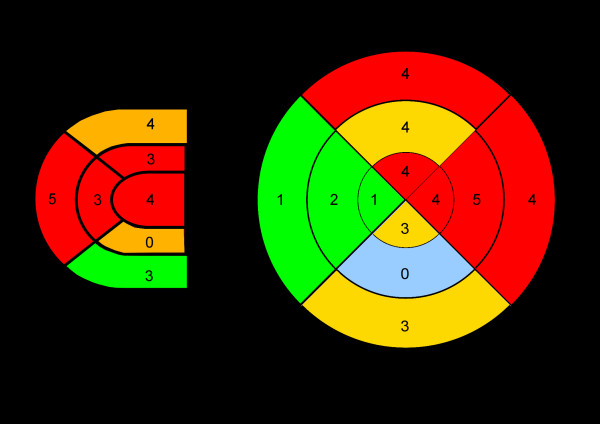
**Distribution of histological inflammation**. Bullseye plots showing distribution of histologically verified inflammation inside right (A) and left (B) ventricular wall from apex (internal segment) to heart base (external segments). Myocardial inflammation was predominantly located in the anterior and lateral wall of right and left ventricle.

In identical slices topography of LGE was classified according to a 7-segment-model of the myocardium (see Figure 1). For each of the 7 segments LGE was noted; if more than one LGE-focus was found for a respective segment all foci were validated. This was done in each of the above mentioned three representative slices. Comparison between LGE-topography and histological topography of inflammation was performed for each CMR-sequence and respective slices.

### Histopathological Analysis

Histopathological analysis was performed by a pathologist blinded to MR-findings. After MR-imaging the heart was explanted by thoracotomy and macroscopy of rats' hearts was evaluated with regards to macroscopic morphology and pericardial effusion according to Yuan Z. et al [16] (Macroscopic morphology: 0 = no inflammatory alteration, 1 = focal inflammatory signs, 2 = multiple/diffuse inflammatory signs ≤1/3 of heart size, 3 = diffuse inflammatory signs >1/3 of heart size, pericardial effusion: 0 = no effusion, 1 = mild effusion, 2 = massive effusion).

Subsequently, hearts were fixed in formalin (4%) and finally embedded in paraffin. In 2 μm thick, Hematoxylin-Eosin (H.E.) stained slices the severity of myocardial inflammation was determined by the following histological criteria according to our previous research [11]:

densitiy of inflammatory infiltration (graded 0-3), strength of inflammatory destruction with rarefaction and pushing-apart of heart muscle fibres (graded 0-3), occurrence of necrosis (graded 0-3), occurrence of pericarditis (graded 0-2). Results were allied into a mircoscopic-score-system, with a maximum score of 11 points, to express severity of myocarditis.

For comparison of LGE-areas with histologically proven areas of inflammation, infiltrated areas were bordered manually in three representative slices from base to apex in a way similar to ROI drawing in CMR images. Area of inflammation was expressed as percentage of the whole slice. Measurement was performed using a dedicated software (analySIS^® ^5.0, Soft Imaging System, Münster, Germany). In identical slices distribution of inflammation was encoded using the same segment model as for LGE-distribution.

### Statistical analysis

All statistical analyses were performed with dedicated software (SPSS^® ^12.0 for Windows, SPSS Inc., Chicago, Illinois, USA).

Percentages of LGE and inflammatory areas relative to healthy myocardial tissue are expressed as means ± standard deviation (SD). Due to non-symmetric distribution of serological parameters (troponin, haproglobin, proBNP) and ordinal scale of macroscopic and histopathological scores we had to use non-parametric statistics on these data. Thus, all correlations were calculated by bivariate correlation analysis using Spearman's correlation coefficient; significant differences of serological parameters between experimental and control group were evaluated by the Mann-Whitney test. Averages; averages for macroscopic and histopathological scores are expressed as median.

In each CMR-sequence with associated slices, four-field-tables, expressing sensitivity, specificity, positive and negative predictive values for LGE, were used to display accuracy of CMR. Histopathological analysis was defined as gold standard.

Results were considered to be significant at p < 0.05.

## Results

All animals of the control group were histopathologically healthy and none revealed LGE by CMR-examination, in contrast all animals of the experimental group (n = 10) had histologically proven myocarditis.

### Comparing topographic distributions of LGE and inflammation

**Figure 2:**
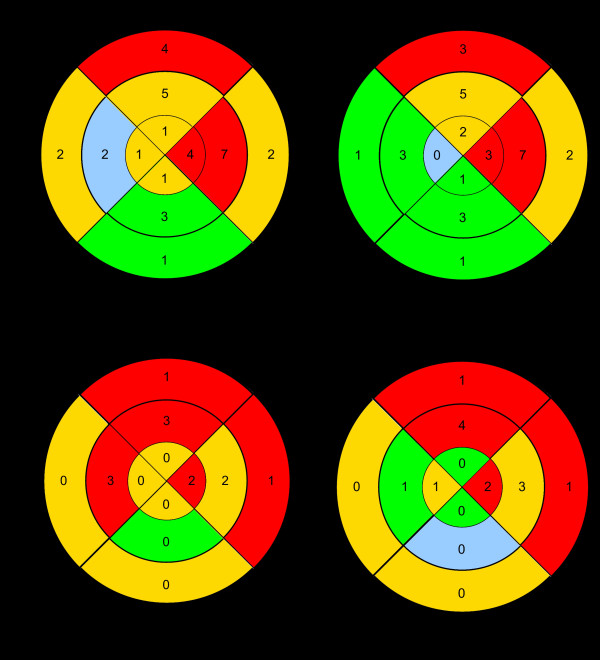
**Distribution of Late Gadolinium Enhancement**. Bullseye plots showing distribution of LGE (LGE) inside the left ventricular wall from apex (internal segments) to heart base (external segments). LGE was mainly located in the anterior and lateral wall of the left ventricle.

**Figure 3:**
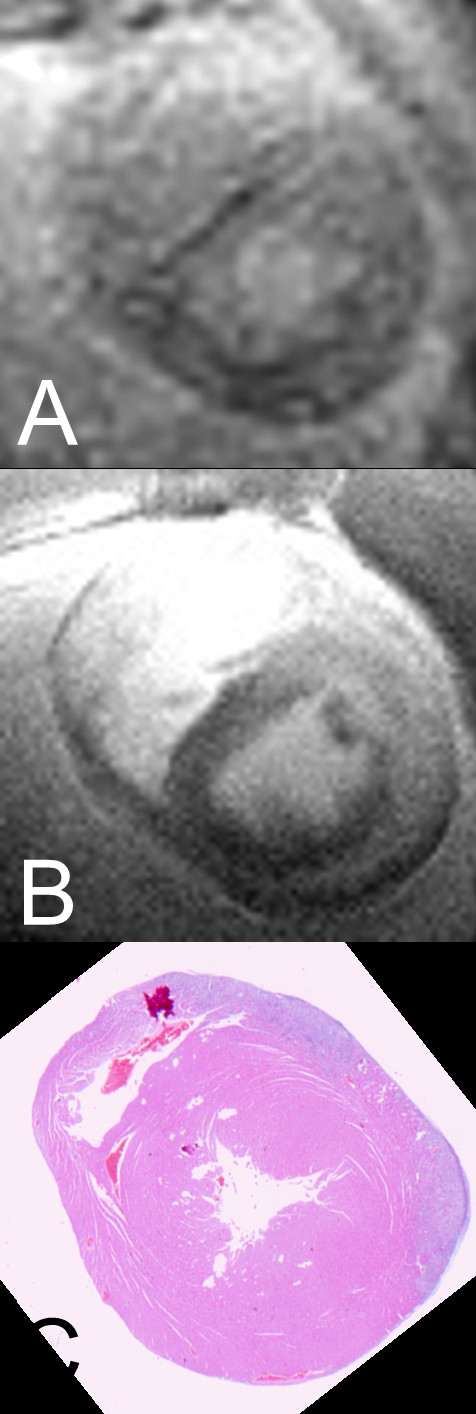
**Sample images of CMR**. CMR images and histopathological finding of an animal from the experimental group: (A) Turbo-FLASH-examination without fat saturation. (B) TSE-examination without fat saturation. (C) Histopathological findings with visible infiltration in the left anterior.

Histologically proven inflammation was predominantly located in the anterior and lateral wall of right and left ventricle (see Figure 1); LGE was found mainly in the anterior and lateral wall of the left ventricle, in no case inside the right ventricular wall (see Figures 2 and 3). Mean percentage of inflammation-areas was 17.82% (SD ± 18.8), mean percentage of LGE-area was 26.12% (SD ± 24.89) in Turbo-FLASH-examination without fat saturation, 22.17% (SD ± 18.17) in Turbo-FLASH-examination with fat saturation, 5.68% (SD ± 7.39) in TSE-examination without fat saturation and 5.42% (SD ± 6.77) in TSE-examination with fat saturation.

Using 4-field-tables we evaluated positive and negative predictive values, sensitivity and specificity of LGE for every examined CMR-sequence (see table 1). As LGE was not observed in the right ventricular wall, those values only refer to the left ventricular wall. In general, results of four-field-tables differed widely due to LGE's location inside the left ventricular wall and examined MR-sequence; LGE located "left anterior" and "left lateral" showed higher values for sensitivity and positive predictive value independent of MR-sequences. Regarding comparison between MR sequences, in TSE examinations sensitivity (maximum 50%) was lower compared to Turbo-FLASH examination (maximum sensitivity 76.92%).

**Table 1:** Results of 4-field-tables

	Sensitivity	Specificity	positive predictive value	negative predictive value
**Turbo-FLASH-examination with fat saturation**				
*left anterior*	61.54%	81.82%	80.0%	64.29%
*left lateral*	69.23%	72.73%	75.0%	66.67%
*left inferior*	0%	73.68%	0.0%	73.68%
*septum*	25.0%	80.0%	20.0%	84.21%
				
**Turbo-FLASH-examination without fat saturation**				
*left anterior*	61.54%	81.82%	80.0%	64.29%
*left lateral*	76.92%	72.73%	76.92%	72.73%
*left inferior*	20.0%	66.67%	12.5%	77.78%
*septum*	0.0%	75.0%	0%	78.95%
				
**TSE-examination with fat saturation**				
*left anterior*	30.77%	81.82%	66.67%	50.0%
*left lateral*	50.0%	90.91%	85.71%	62.5%
*left inferior*	0%	100.0%	0	79.17%
*septum*	0%	90.0%	0	81.82%
				
**TSE-examination without fat saturation**				
*left anterior*	25.0%	90.91%	75.0%	52.63%
*left lateral*	15.38%	72.73%	40.0%	42.11%
*left inferior*	0%	100%	0%	79.17%
*septum*	25.0%	90%	33.33%	85.71%

Results of 4-field-tables for LGE detected by different CMR-sequences.

In Turbo -FLASH-examination (without and with fat saturation) sensitivity and positive predictive value were best "left anterior" and "left lateral" (sensitivity: 61.54%-76.92%; positive predictive value: 75%-80%; see tab. 1) compared to "left inferior" and "septum" (sensitivity: 0%-25%; positive predictive value: 0%-20%; see tab. 1). Specificity and negative predictive value hardly varied depending on location of LGE (specificity: 66.67%-81.82%; negative predictive value: 64.29%-84.21%; see tab. 1).

Regarding TSE-examination (without and with fat saturation) sensitivity and positive predictive value were highest if LGE was located "left anterior" and "left lateral" (sensitivity: 15.38%-50%; positive predictive value: 40.0%-85.71%; see tab.1). Negative predictive value was best "left inferior" and "septum" (79.17%-85.71%; see tab. 1) compared to "left anterior" and "left lateral" (42.11%-62.5%; see tab.1). Comparable to Turbo-FLASH-examination, specificity was almost stable independent of LGE's location (72.73%-100%; see tab. 1).

### Correlation between CMR and serology

Animals in experimental group had significantly higher serological levels of haptoglobin and troponinT on day 21 compared to animals of control group (see table 2: Mann-Whitney test). ProBNP values were constant in experimental and control group on days 1, 7 and 21, thus a correlation between the extent of LGE and proBNP could not be calculated.

**Table 2:** Comparison of serological parameters in control and experimental group

	mean rank	
	***control group***	***experimental group***

**Haptoglobin**		
*day 1*	10.5	10.5
*day 7*	10.3	10.7
*day 21*	9.2	11.8
		
**troponinT**		
*day 1*	10.5	10.5
*day 7*	10.0	11.0
*day 21*	5.5	15.5
		
**proBNP**		
*day 1*	10.5	10.5
*day 7*	10.5	10.5
*day 21*	9.5	9.5

Mann-Whitney test for serological levels of haptoglobin, troponinT and proBNP, measured on days 1, 7 and 21 in control and experimental group.

Correlations between the size of LGE and troponinT-levels of day 21 were found (see table 3). LGE-areas determined in Turbo-FLASH-sequences as well as in TSE-sequences with and without fat saturation correlated highly with troponin-levels measured on day 21 (Turbo-FLASH without fat saturation: r = 0.64, p < 0.05; Turbo-FLASH with fat saturation r = 0.75, p < 0.05; TSE without fat saturation r = 0.95, p < 0.05; TSE with fat saturation: r = 0.75, p < 0.05).

**Table 3:** Correlation between LGE and serological parameters

	Turbo-FLASH	Turbo-FLASH with fs	TSE	TSE with fs
**troponinT**	r = 0.64; p < 0.05	r = 0.75; p < 0.05	r = 0.95; p < 0.05	r = 0.76; p < 0.05
**haptoglobin**	r = 0.11; p = 0.80	r = 0.00; p = 1.0	r = -0.23; p = 0.59	r = 0.16; p = 0.70

Spearman's correlations between extend of LGE and serological markers measured on day 21. As levels of proBNP were constant, correlations between LGE and proBNP-levels could not be calculated.

With regards to serum-levels of haptoglobin we found no correlation with extent of LGE (see table 3).

### Correlation between CMR and histological severity of myocarditis

Pericardial effusion as well as visible signs of inflammation were found in nine of ten animals of the experimental group, the median macroscopic score was 3 (range 0-5).

A high density of inflammatory infiltration with a subepicardial accentuation in combination with destructed heart muscle fibres that were pushed apart was a typical histological finding in the experimental group; the median microscopic score was 9 (range 1-11). All animals of the control group presented with histologically healthy myocardial tissue.

The extent of LGE-areas measured in Turbo-FLASH-sequences correlated highly with the histopathological severity of myocarditis (r = 0.94, p < 0.01 for Turbo-FLASH; r = 0.96, p < 0.01 for Turbo-FLASH with fat saturation; see table 4); Correlations between TSE-examination and histological severity missed statistical significance (r = 0.67, p = 0.06 TSE with fat saturation; r = 0.43, p = 0.29 TSE; see table 4) as their p-values were above 0.05.

**Table 4:** Correlation between extend of LGE and histopathological score

	Turbo-FLASH	Turbo-FLASH with fs	TSE	TSE with fs
**histopathological score**	r = 0.94; p < 0.05	r = 0.96; p < 0.05	r = 0.43; p = 0.29	r = 0.68; p = 0.06

Spearman's correlations between extend of LGE measured in different CMR-sequences and histopathological score. (Turbo-FLASH = gradient-echo-sequence; TSE = turbo-spin-echo-sequence; fs = fat saturation).

## Discussion

Myocarditis is defined as myocardial inflammation presenting with edema, cellular infiltration, apoptosis and necrosis of cardiomyocytes[17]. In up to 20% it accounts for postinflammatory dilated cardiomyopathy in children[18,19]. It is mostly diagnosed clinically, however in some cases its diagnosis has to be verified by Endomyocardial Biopsy (gold standard). Due to a diffuse to focal inflammatory pattern the sampling error results in low sensitivity of EMB[2]. Usually five biopsies are taken from myocardium; increasing the number of biopsies to 17 per patient could improve sensitivity up to 80%[20] however this is associated with increased invasiveness. Instead, in some centers biopsies are taken from myocardium that presented as LGE to improve sensitivity[3,4].

### Pathophysiology of LGE

LGE has been established in diagnosing myocarditis in the past[4,21-23]. Hypotheses concerning pathophysiology of LGE in myocarditis have been made for a long time. Necrotic cardiomyocytes, as found in animals of experimental group, with ruptured membranes could be able to take up contrast media such as Gadolinium-DTPA, whereas Gadolinium as an extracellular molecule cannot penetrate into healthy cardiomyocytes, resulting in contrast media enhancement of necrotic foci. Abdel-Aty et al.[24] explained this process during their study of differentiating acute and chronic myocardial infarction that as being related to the increased volume of distribution of gadolinium chelates secondary to extracellular space expansion in myocardial scars. The underlying mechanism of this extracellular space enlargement, however, differs in relation to infarct age. In acute myocardial infarction, there is loss of membrane integrity of the already edematous cardiomyocytes, allowing communication between the extracellular and intracellular spaces. Moreover, the induction of reperfusion marks the rapid evolution of an inflammatory-like response of which interstitial edema is a substantial feature, even though edema does not seem case LGE. In chronic myocardial infarction, on the other hand, enlargement of the extracellular space is mostly the result of the relatively large collagen matrix in the absence of myocardial edema. These considerations explain that LGE directly correlated to the extent of myocardial cell necrosis and this is a feature of myocardial necrosis regardless of its acuity or chronicity. This conclusion is also agreed by Friedrich et al. [25].

### Correlations between CMR and serology

TroponinT seems to have a correlation with the size of LGE. In all measured MR sequences we found statistically significant positive correlations (Turbo-FLASH without fat saturation: r = 0.64, p < 0.05; Turbo-FLASH with fat saturation r = 0.75, p < 0.05; TSE without fat saturation r = 0.95, p < 0.05; TSE with fat saturation: r = 0.75, p < 0.05; see table 3). Being dependent on the degree of cellular necrosis, troponin level is directly correlated to the extent of LGE.

Even though animals of the experimental group had significantly higher haptoglobin levels on days 7 and 21, haptoglobin had no correlation to the extent of LGE. Pathologcially raised levels of haptoglobin in control group can be explained by the unspecific inflammation caused by Complete Freud's Adjuvance injected in control animals.

### Correlation between CMR and histological severity of myocarditis

As our results demonstrate, there is a statistically significant correlation between the extent of LGE measured in Turbo-FLASH-sequences and the histopathological severity of inflammation expressed as histopathological score. This stands in contrast to the results of Gutberlet et al, who showed that the inflammatory activity as verified by histopathology does not correlate well with the extent of LGE [26].

### Comparing topographic distributions of LGE and inflammation

LGE was found exclusively inside the left ventricular wall, despite cardiac lesions of the right ventricle found in histology. Probably, because the rat's heart is of small size, the right ventricular wall with its fewer muscular mass compared to the left ventricle could not be displayed properly by CMR-examination. Thus identifying possible LGE inside the right ventricle failed.

As described in results, sensitivity as well as positive predictive values varied widely dependending on the location of LGE and examined CMR-sequence. In general, inflammations located in the left anterior and lateral ventricular wall were identified by Turbo-FLASH-sequences in 61.54-76.92% whereas sensitivity dropped to 0-20% for inflammations located in the left inferior myocardium and in the septum; LGE located in the left anterior and lateral ventricular wall had high positive predictive values (75%-80%) in Turbo-FLASH-examination. Possible hypotheses for dependence on location of LGE (positive predictive value) and inflammation (sensitivity) could be: We believe that in Experimental Autoimmune myocarditis inflammation occurs primarily in the left anterior and lateral ventricular wall and delayed in the left inferior myocardium and septum and thus on day 21 inflammation could have been on different stages inside the myocardium as proved by histology. Cardiac lesions located "left inferior" and "septum" were of small size and only found in three animals. As it is challenging to compare histological segments with exactly corresponding MR-slices, we think that finding cardiac lesions with a small extent (e.g. located *left inferior *and *septum*) by MR examination is more difficult and resulted in low sensitivity. On the other hand it is possible that myocardial inflammation located in the left inferior ventricular wall and in the septum had caused mainly extracellular edema and hardly histologically verifiable myocardial damage as found in histology. In consequence raised interstitial space could lead to LGE without a histologically verifiable, corresponding myocardial inflammation, resulting in low positive predictive values. Furthermore, technical issues such as field inhomogenity of reception coil and motion artefacts could have an influence on our described results and might lead to differences in accuracy of CMR for different myocardial locations.

In their study Mahrholdt et al.[23] could show a sensitivity for LGE in diagnosing acute viral myocarditis of 95%. In this study LGE was compared to histological findings of CMR-guided Endomyocardial Biopsy. In our study we compared LGE to histopathological analysis of the whole myocardium, which consequently has a higher sensitivity than EMB. This explains lower values for sensitivity in our study. Nevertheless we found higher sensitivity (up to 76%) for the locations "left anterior" and "left lateral" compared to EMB.

In another study Mahrholdt et al.[4] were able to increase sensitivity of EMB by taking biopsy close to contrast enhanced myocardium. With our results for positive predictive value (up to 80%) we agree, that EMB should be performed "CMR-guided". Nevertheless, clinical studies should investigate, if sensitivity and positive predictive value vary depending on location of LGE and histological inflammation in humans, too.

Adequate histopathological criteria predicting progression into chronic disease or death are missing[27] and further, using Dallas criteria often results in false-negative cases of myocarditis[28]. Concluding, diagnostic information delivered by EMB seems to be reduced to displaying myocardial inflammation. Taking biopsies from myocardium that presents with contrast enhancement is described to be easier for the right ventricle but appears complex for the left ventricular wall. We were able to show that histological severity of myocarditis-induced injury correlates with the regional distribution and extent of LGE. Accordingly, CMR seems to provide similar diagnostic information (unmasking myocardial inflammation, its distribution and information about severity) as EMB. Kuhn et al.[29] see no indication for EMB in routinely diagnosing myocarditis and at least we think that indication for EMB should be revised.

Munk et al.[30] mentioned that troponin T levels below 0.1 μg/l predict absence of contrast enhancement in acute phase of myocarditis. In this aspect the current study results regarding the correlation between troponin T and size of LGE agree with Munk et al.

### Study limitations

Differences in slice thickness of histopathological (2 μm) and MR examination (3 mm) has to be seen critically. As the histologically proven area of inflammation stretched across several slices, we do not think that differences in slice thicknesses had a strong influence on data analyses. Another limitation of the study is that criteria used for evaluation is center specific which may be difficult to put the results into perspective with other reports. We did not use Dallas criteria as their use often results in false-negative cases of myocarditis and they seem to be no predictor of outcome in myocarditis[31]; instead we used above mentioned criteria which are standard in our department of pathology and were used in our previous research [11].

The current study is based on an animal model for acute autoimmune myocarditis. In humans acute myocarditis is mostly caused by viral infection. However, in our animal model it is possible to perform CMR-examination and histolgical analysis of the whole myocardium on the same day. As we wanted to show the reliability of CMR in showing topography of myocardial inflammation, we do not think that different aetiologies (autoimmune in the current study vs. viral in humans) inhibits transfer of current study results to humans.

Using a 1.5T clinical scanner in combination with clinical MR-sequences has to be seen as a limitation regarding spatial resolution. However most centers use 1.5T scanners, thus we think, in this way our result can be compared to further studies more easily. Finally, we did not provide data about early enhancement, nor about T2-weighed images as proposed by Friedrich et al [32].

## Conclusions

We were able to show that EAM as a model of human myocarditis can be detected by clinical CMR providing the opportunity for further fundamental research. The extent of LGE correlates to the histological severity of myocarditis and to serum-levels of troponinT on day 21. Areas of LGE have nearly identical topographic distribution as compared to histologically proven areas of inflammation. Late gadolinium enhancement seems to depict cardiomyocytes necrosis and thus should be regarded as a marker indicating more severe myocarditis. In conclusion, the accuracy of CMR in diagnosing myocarditis is high enough to guide endomyocardial biopsy.

## Competing interests

The authors declare that they have no competing interests.

## Authors' contributions

HK conceived the study and together with PE carried out the induction of myocarditis as well as MRI- measurement and -analysis. FH adapted MR-sequences to the animal conditions. RB performed histopathological analysis. HA accomplished together with HK and PE statistical analysis. TJV was involved in drafting the manuscript and revised it critically. All authors read and approved the final manuscript.
